# Tris(1,3-dichloro-2-propyl) phosphate disrupts dorsoventral patterning in zebrafish embryos

**DOI:** 10.7717/peerj.4156

**Published:** 2017-12-14

**Authors:** Subham Dasgupta, Sara M. Vliet, Allison Kupsco, Jessica K. Leet, Diego Altomare, David C. Volz

**Affiliations:** 1 Department of Environmental Sciences, University of California, Riverside, CA, United States of America; 2 Environmental Toxicology Graduate Program, University of California, Riverside, CA, United States of America; 3 University of South Carolina, Columbia, SC, United States of America

**Keywords:** TDCIPP, Zebrafish, Dorsoventral patterning, Embryo, Flame retardant, Public health

## Abstract

Tris(1,3-dichloro-2-propyl) phosphate (TDCIPP) is a high-production volume organophosphate flame retardant widely used within the United States. Within zebrafish, initiation of TDCIPP exposure at 0.75 h post-fertilization (hpf) results in genome-wide alterations in methylation during cleavage (2 hpf) as well as epiboly delay or arrest (at higher concentrations) during late-blastula and early-gastrula (4–6 hpf). To determine whether these TDCIPP-induced effects were associated with impacts on the transcriptome, embryos were exposed to vehicle (0.1% DMSO) or 2 µM TDCIPP from 0.75 hpf to 6 hpf, and total RNA was extracted from triplicate embryo pools per treatment and hybridized onto duplicate Affymetrix Zebrafish Gene 1.0 ST Arrays per RNA sample. Based on transcriptome-wide profiling, TDCIPP resulted in a significant impact on biological processes involved in dorsoventral patterning and bone morphogenetic protein (BMP) signaling. Consistent with these responses, TDCIPP exposure also resulted in strongly dorsalized embryos by 24 hpf—a phenotype that mimicked the effects of dorsomorphin, a potent and selective BMP inhibitor. Moreover, the majority of dorsalized embryos were preceded by epiboly arrest at 6 hpf. Our microarray data also revealed that the expression of sizzled (*szl*)—a gene encoding a secreted Frizzled-related protein that limits BMP signaling—was significantly decreased by nearly 4-fold at 6 hpf. Therefore, we used a splice-blocking morpholino to test the hypothesis that knockdown of *szl* phenocopies TDCIPP-induced delays in epiboly progression. Interestingly, contrary to our hypothesis, injection of *szl* MOs did not affect epiboly progression but, similar to *chordin* (*chd*) morphants, resulted in mildly ventralized embryos by 24 hpf. Overall, our findings suggest that TDCIPP-induced epiboly delay may not be driven by decreased *szl* expression, and that TDCIPP-induced dorsalization may—similar to dorsomorphin—be due to interference with BMP signaling during early zebrafish development.

## Introduction

Tris (1,3-dichloro-2-propyl) phosphate (TDCIPP) is an organophosphate flame retardant extensively used in polyurethane foam, acrylic latexes, plastics and resins (WHO, 1998). As such, TDCIPP has been detected at elevated levels within consumer products and indoor dust, resulting in TDCIPP exposure within pediatric populations (Butt et al., 2014; Stapleton et al., 2012). For example, TDCIPP was detected in 100% of residential dust samples (mean concentration = 1,390 ng/g) and hand wipes (mean concentration = 84.1 ng/hand wipe) collected from children; TDCIPP concentrations were also associated with levels of bis(1,3-dichloro-2-propyl) phosphate (BDCIPP, the primary metabolite of TDCIPP) in urine samples, suggesting that hand-to-mouth transfer and/or dermal absorption may be possible routes of exposure of TDCIPP within children (Hoffman et al., 2015). Indeed, urinary BDCIPP levels in children who washed hands frequently were at least 60% lower than children with less frequent washes (Butt et al., 2014). TDCIPP has also been detected in nearly 50% of placental samples (maximum concentration = 83 ng/g lipid weight) collected from 50 women in Eastern China (Ding et al., 2016). Although TDCIPP and/or BDCIPP has been detected within human populations around the world, our understanding about the potential effects of TDCIPP during early vertebrate development is relatively limited.

Using zebrafish as a model, several recent studies have revealed that TDCIPP induces a variety of abnormal phenotypes at different stages of embryonic development. For example, embryos exposed to 3 µM TDCIPP starting at 0.75 h post-fertilization (hpf) (2-cell stage) resulted in significant mortality and developmental abnormalities at 96 hpf (McGee et al., 2012). However, no significant malformations were observed following initiation of 3 µM TDCIPP exposure at 5.25 hpf (50% epiboly), suggesting that cleavage and/or blastula stages of development represent sensitive windows for TDCIPP-induced developmental toxicity (McGee et al., 2012). Indeed, initiation of TDCIPP exposure at 0.75 hpf resulted in a concentration-dependent delay or arrest in epiboly during blastula and gastrula (4–6 hpf) (Fu et al., 2013; Kupsco et al., 2017). Epiboly delays at ∼6 hpf also occurred in unexposed F1 embryos following TDCIPP-exposed F0 parents, an effect that was likely due to maternal transfer into F1 embryos (Yu et al., 2017).

Importantly, these studies demonstrate that TDCIPP has the potential to adversely impact early developmental processes both through direct exposure and/or maternal transfer. Therefore, using microarray-based expression profiling, the first objective of this study was to determine whether TDCIPP-induced epiboly delays following initiation of exposure at 0.75 hpf are associated with impacts on the transcriptome during cleavage (2 hpf) and gastrula (6 hpf); these two stages flank the maternal-to-zygotic transition (MZT, ∼3 hpf), a period characterized by rapid maternal transcript degradation and zygotic genome activation (Tadros & Lipshitz, 2009). Using a combined genetic and pharmacologic strategy, the second objective of this study was to determine whether TDCIPP-induced impacts on epiboly—as well as potential targets identified by microarray-based expression profiling—are associated with downstream effects on dorsoventral patterning during segmentation.

## Materials and Methods

### Animals

Adult wildtype (5D) zebrafish were maintained and bred on a recirculating system using previously described procedures (Vliet, Ho & Volz, 2017); to establish our fish colony, specific pathogen-free (SPF) 5D zebrafish were originally purchased from Dr. Robert Tanguay (Oregon State University). Adult breeders were handled and treated in accordance with an Institutional Animal Care and Use Committee (IACUC)-approved animal use protocol (#20150035) at the University of California, Riverside.

### Chemicals

TDCIPP (99% purity) and dorsomorphin (DMP) (99.7% purity) were purchased from ChemService and Sigma-Aldrich, respectively. For both chemicals, stock solutions were prepared in high performance liquid chromatography (HPLC)-grade dimethyl sulfoxide (DMSO), and stored within 2-mL amber glass vials with polytetrafluoroethylene-lined caps. Working solutions were prepared in embryo media (5 mM NaCl, 0.17 mM KCl, 0.33 mM CaCl_2_, 0.33 mM MgSO_4_, pH 7) immediately prior to each experiment.

### TDCIPP and dorsomorphin exposures

All embryos were staged according to previously described methods (Kimmel et al., 1995). Viable 5D embryos at the 2-cell stage (0.75 hpf) were exposed to 10 mL of vehicle (0.1% DMSO), TDCIPP (1.56 µM and 3.12 µM) or dorsomorphin (0.078 µM, 0.156 µM, 0.312 µM, and 0.625 µM) in clean 60-mm glass petri dishes. Final dorsomorphin concentrations were selected based on initial range-finding exposures that relied on survival and severity of dorsalization as endpoints. All exposures were conducted at 28 °C within a temperature-controlled incubator under a 14-h:10-h light:dark cycle. At 6 and 24 hpf, petri dishes were removed from the incubator, coagulated embryos discarded, and live embryos were imaged under transmitted light using a Leica MZ10 F stereomicroscope (Leica Microsystems, Inc., Buffalo Grove, IL, USA) equipped with a DMC2900 camera. The presence or absence of epiboly delay or arrest was assessed at 6 hpf, and the severity of dorsalization at 24 hpf was determined according to previously described classifications (Cannon et al., 2010; Kishimoto et al., 1997).

### Transcriptome profiling using zebrafish-specific microarrays

Viable 5D embryos were exposed to 10 mL of vehicle (0.1% DMSO) or 2 µM TDCIPP in triplicate glass beakers under a 14-h:10-h light:dark cycle and static conditions at 28°C from 0.75 hpf to 2 or 6 hpf. The concentration of TDCIPP (2 µM) was selected based on our earlier studies (McGee et al., 2012; Volz et al., 2016), and exposure to this concentration results in a similar magnitude of toxicity at 6 and 24 hpf compared to embryos exposed to 1.56 µM TDCIPP (one of the concentrations used for epiboly and dorsalization assessments in this study). Embryos (25 per replicate) were collected at either 2 or 6 hpf, transferred from beakers to 2-mL cryovials, snap-frozen in liquid nitrogen, and stored at −80 °C. These experiments resulted in three independent replicate samples for each time point and treatment group, resulting a total of 12 samples for RNA extractions. Total RNA was extracted from pooled embryos using a SV Total RNA Isolation System (Promega, Madison, WI, USA). After elution of RNA in 100 µL nuclease-free water, total RNA concentrations, 260/280 ratios, and 260/230 ratios were quantified using a NanoDrop ND-2000 spectrophotometer (Thermo Fisher Scientific, Waltham, MA, USA) and then stored at −80 °C; total RNA quality was also confirmed using an Agilent 2100 Bioanalyzer system.

Total RNA samples were amplified and biotinylated using GeneChip WT PLUS Reagent Kit (Affymetrix). Briefly, 100 ng of total RNA per sample was reverse-transcribed into ds-cDNA using NNN random primers containing a T7 RNA polymerase promoter sequence; cDNA quality was then confirmed using an Agilent 2100 Bioanalyzer system. T7 RNA polymerase was then added to cDNA samples to amplify RNA, and then RNA was reverse-transcribed to ss-DNA and degraded using RNase H. ss-DNA molecules were then fragmented and terminally labelled with biotin. Amplified and labeled samples were hybridized onto duplicate Zebrafish Gene 1.0 ST Arrays (Affymetrix, Santa Clara, CA, USA) for 16 h at 45 °C using a GeneChip Hybridization Oven 640 and a GeneChip Hybridization, Wash, and Stain Kit (Affymetrix); these arrays were constructed by Affymetrix based on the danRer6/Zv9 genome build and contained 1,255,682 probes representing 59,302 unique genes. Hybridized arrays were washed and stained using GeneChip Fluidics Stations 450 (Affymetrix). Arrays (24 total) were then scanned using a GeneChip Scanner 3000 7G system and computer workstation equipped with GeneChip Command Console 4.0 software (Affymetrix).

Following completion of array scans, probe cell intensity (CEL) files (24 total) were imported into Expression Console Software (Affymetrix) and processed at the gene-level using Affymetrix’s ZebGene-1_0-st library file and Robust Multichip Analysis (RMA) algorithm to generate CHP files; all CEL and CHP files are available for download via NCBI’s GEO DataSets database under Accession ID GSE106875. After confirming data quality within Expression Console, CHP files containing log2 expression signals for each probe were then imported into Transcriptome Analysis Console Software (Affymetrix) and R (http://www.r-project.org) to analyze treatment-specific transcriptional responses using volcano plots and one-way between-subject analysis of variance (ANOVA). Affymetrix transcript cluster IDs for differentially expressed genes with False Discovery Rates (FDR) of *p* < 0.1 were imported into DAVID Bioinformatics Resources 6.8 for Gene Ontology (GO) enrichment analysis against zebrafish genome assembly GRCz10. DAVID-based analysis was performed on biological process (GOTERM_BP_FAT) using the functional annotation tool and Fisher exact *p*-value as the parameter for statistical significance (Xu et al., 2017).

### Morpholino knockdowns

Morpholino (MO) antisense oligos were synthesized and obtained from Gene Tools, Inc. Based on our microarray data, a fluorescein-tagged splice-blocking MO was designed to target the first exon-intron boundary (E1I1) of the zebrafish *sizzled* (*szl*) gene (NCBI Gene ID: 353294), leading to insertion of intron 1 within *szl* mRNA (*szl*-MO sequence: 5′-ggtgtctcaacacgtacctgtcgag-3′). Gene Tools’ standard fluorescein-tagged negative control MO (nc-MO) was used in order to account for potential non-target MO toxicity, and a zebrafish-specific, fluorescein-tagged *chordin* MO (*chd*-MO sequence: 5′-atccacagcagcccctccatcatcc-3′) was used a positive control. MO stock solutions (10 mM) were prepared by resuspending lyophilized MOs in molecular-biology grade (MBG) water, and stocks were stored at room temperature in the dark.

On the same day as injections, working solutions of nc-MOs and *szl*-MOs were diluted to 5 mM in MBG water, and working solutions of *chd*-MOs were diluted to 1.25 mM in MBG water. Newly fertilized (1- to 8-cell stage, or before 1.25 hpf) wildtype (5D) zebrafish embryos were microinjected with MOs (∼3 nL per embryo) using a motorized Eppendorf Injectman NI2 and FemtoJet 4× similar to previously described protocols (McGee et al., 2013). At 3 hpf, MO delivery in embryos was confirmed using a Leica MZ10 F stereomicroscope equipped with a DMC2900 camera and a GFP filter cube; non-fluorescent and/or coagulated embryos were discarded. Embryos were imaged at 6 and 24 hpf as described above.

To confirm *szl* knockdown, embryos (20 per injection pool) were snap-frozen in liquid nitrogen at 6 hpf and stored at −80 °C. Total RNA was extracted as described above and eluted in 25 µL of nuclease-free water. RNA quality and quantity was confirmed using an Agilent 2100 Bioanalyzer system and Qubit 2.0 Fluorometer (Thermo Fisher Scientific), respectively. A total of ∼140 ng RNA per sample was reverse-transcribed into ds-cDNA using a GoScript Reverse Transcription System (Promega). An E1:E2 *szl* fragment (∼455 bp) was then amplified (forward primer: 5′-gtcttcagtgatgtctctattcagtctg-3′; reverse primer: 5′-ctttggagaaagcactgatgtgttt-3′) in triplicates using approximately 50 ng of cDNA per sample, ZymoTaq PreMix (Zymo Research, Irvine, CA, USA), and an Eppendorf Mastercycler Nexus Thermocycler with the following conditions: 2 min at 95 °C followed by 45 cycles of 95 °C for 30 s, 49 °C for 1 min, and 72 °C for 30 s. PCR products were visualized using an Agilent 2100 Bioanalyzer system, or 1% agarose gels stained with either ethidium bromide or Bio-Rad UView dye. All agarose gels were imaged using a Bio-Rad Gel Doc System.

## Results

### TDCIPP disrupts biological processes specific to BMP signaling and dorsoventral patterning

**Figure 1:**
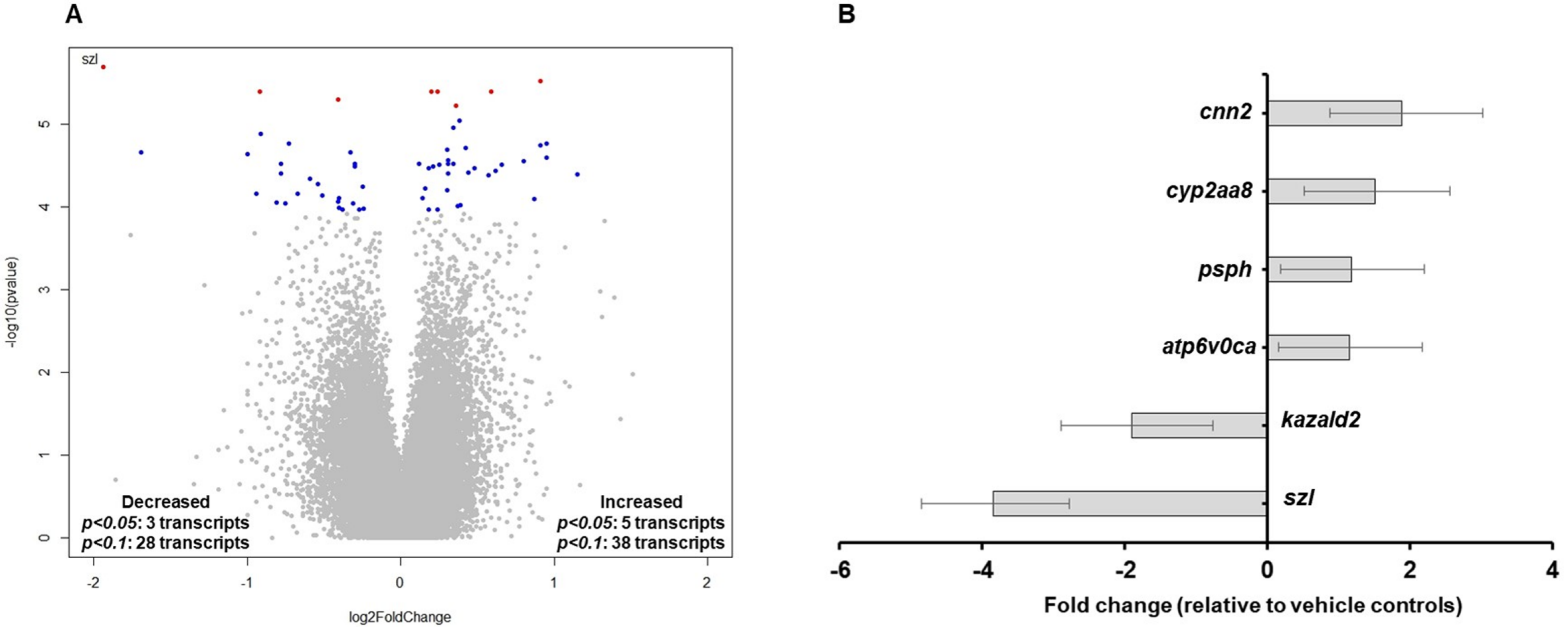
TDCIPP disrupts biological processes that regulate BMP signaling and dorsoventral patterning. (A) Volcano plot showing transcriptome-wide responses following exposure to 2 µM TDCIPP from 0.75 to 6 hpf. *x* axis = log2 (fold-change); *y* axis = −log10 (*p*-value). Blue and red dots denote genes with a FDR *p* < 0.1 and *p* < 0.05, respectively. (B) Annotated transcripts with statistically significant fold changes (FDR *p* < 0.05) following exposure to 2 µM TDCIPP from 0.75 to 6 hpf. Data are presented as mean fold-change ± standard deviation relative to vehicle controls. N, three independent replicate samples per treatment group and two duplicate arrays per replicate sample.

Based on transcriptome profiling using zebrafish-specific microarrays, initiation of TDCIPP exposure at 0.75 hpf resulted in no significant impacts on transcript levels at 2 hpf (pre-MZT) (Fig. S1). However, at 6 hpf (post-MZT), a total of 8 and 66 transcripts were significantly affected based on a FDR *p*-value of <0.05 and <0.1, respectively (Fig. 1A). Figure 1B shows fold changes of the six most significantly affected and annotated transcripts (FDR *p* < 0.05), where *sizzled* mRNA (*szl*, NCBI Gene ID: 353294) was the most impacted with an approximately 4-fold decrease relative to vehicle controls. GO enrichment analysis based on 42 DAVID-identified transcripts with FDR *p*-values < 0.1 demonstrated that the three most significant biological processes affected by TDCIPP exposures were specific to BMP signaling and dorsoventral patterning (Table 1).

**Table 1:** Zebrafish-specific Gene Ontology (GO) enrichment analysis based on transcripts significantly affected (FDR *p* < 0.1) following exposure to 2 µM TDCIPP from 0.75 to 6 hpf.

GO term	Gene count	Genes (fold change)	Fisher’s exact *p* value
Regulation of BMP signaling pathway	3	*bambia* (−1.72), *chd* (+1.58), *szl* (−3.85)	3.1E−5
Somitogenesis	4	*tbx16* (−1.21), *chd* (+1.58), *foxb1a* (−1.92), *igf2a* (−1.92)	3.8E−5
Dorsal/ventral pattern formation	4	*bambia* (−1.72), *bmpr1aa* (−1.43), *chd* (+1.58), *szl* (−3.85)	1.1E−4
Heart looping	4	*tbx16* (−1.21), *apobec2a* (−1.18), *bmpr1aa* (−1.43), *chd* (+1.58)	1.1E−4
Regulation in BMP signaling pathway involved in heart jogging	2	*tbx16* (−1.21), *chd* (+1.58)	3.3E−4

### TDCIPP-exposed embryos phenocopy dorsomorphin (DMP)-exposed embryos by 24 hpf

**Figure 2:**
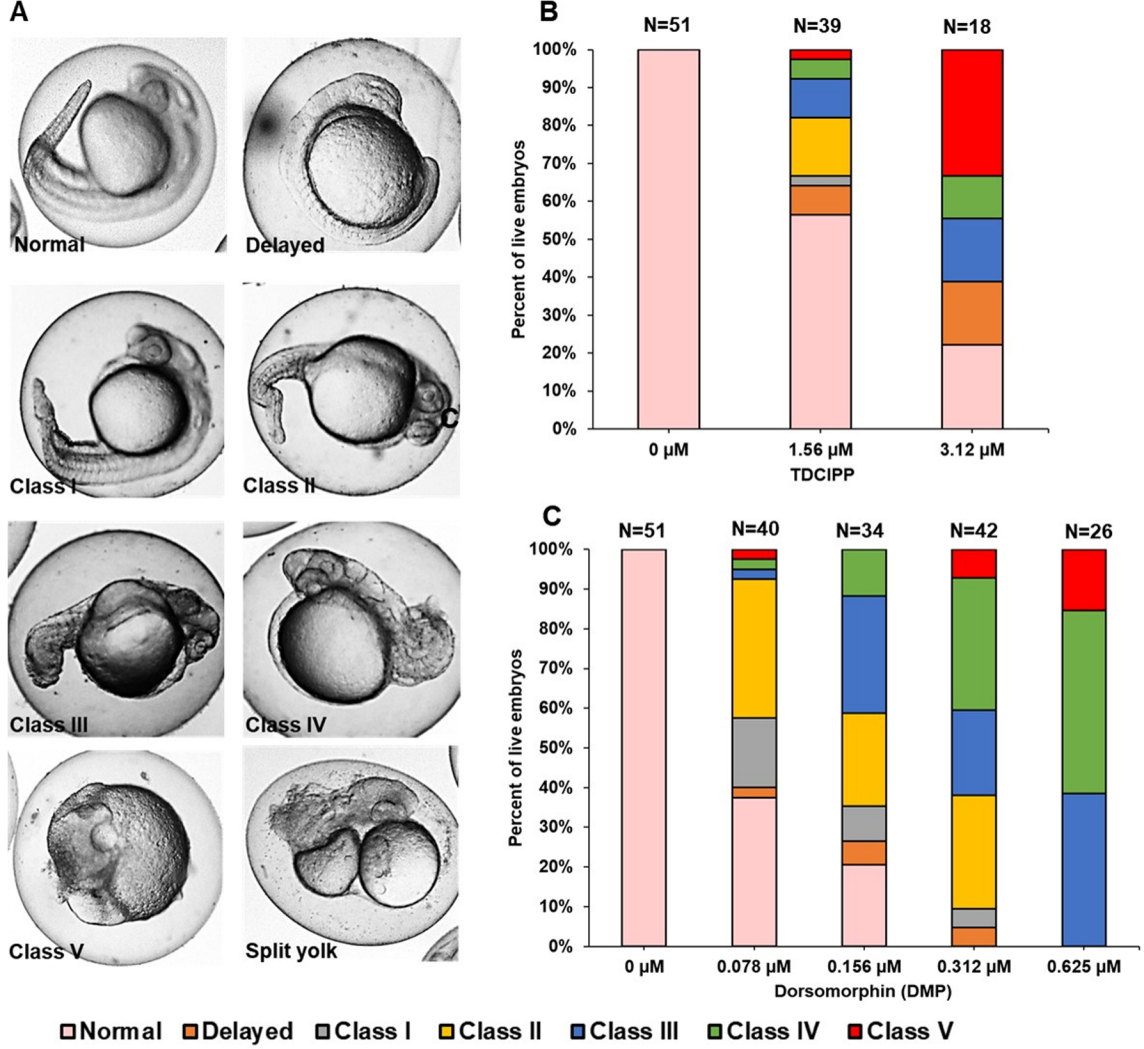
TDCIPP-exposed embryos phenocopy embryos exposed to dorsomorphin from 0.75 to 24 hpf. (A) Representative 24-hpf images of normal embryos, delayed embryos, Class I–V levels of dorsalization, and split yolk embryos following exposure to TDCIPP or DMP. (B) Distribution of dorsalization classes following exposure to TDCIPP from 0.75 to 24 hpf. (C) Distribution of dorsalization classes following exposure to DMP from 0.75 to 24 hpf. Depending on the TDCIPP concentration (1.56 or 3.12 µM), the severity of dorsalization varied from mild (Class I) to strong (Class V).

To confirm whether TDCIPP-induced effects on BMP-related pathways at 6 hpf led to alterations in dorsoventral patterning at 24 hpf, we exposed 0.75 hpf embryos to TDCIPP (1.56 and 3.12 µM) or DMP (0.078–0.625 µM), a BMP pathway inhibitor that induces dorsalization within zebrafish embryos (Cannon et al., 2010). Dorsalized phenotypes were categorized into five classes based on the severity of effect, ranging from Class I (only the ventral tail fin is absent) to Class 5 (posterior and ventral structures completely fail to develop) (Kishimoto et al., 1997) (Fig. 2A). Exposure to TDCIPP from 0.75 to 24 hpf resulted in a concentration-dependent increase in dorsalized phenotypes, with 36% and 61% of embryos being dorsalized following exposure to 1.56 and 3.12 µM, respectively (Fig. 2B). Among the dorsalized embryos, there was a concentration-dependent increase in the severity of dorsalization, with 8% and 61% of embryos showing strong dorsalization (Classes IV–V) following exposure to 1.56 and 3.12 µM, respectively. The observed phenotypes and classes of TDCIPP-induced dorsalization were comparable to embryos exposed to DMP from 0.75 to 24 hpf, which also demonstrated a concentration-dependent increase in the severity of dorsalization (Fig. 2C). While rare, dorsalized embryos following exposure to TDCIPP also resembled a split-yolk phenotype (<10% of TDCIPP-exposed embryos) (Fig. 2A). In addition, a small proportion of embryos (8–17%) were developmentally delayed by ∼6–8 h relative to vehicle (0.1% DMSO) controls.

**Figure 3:**
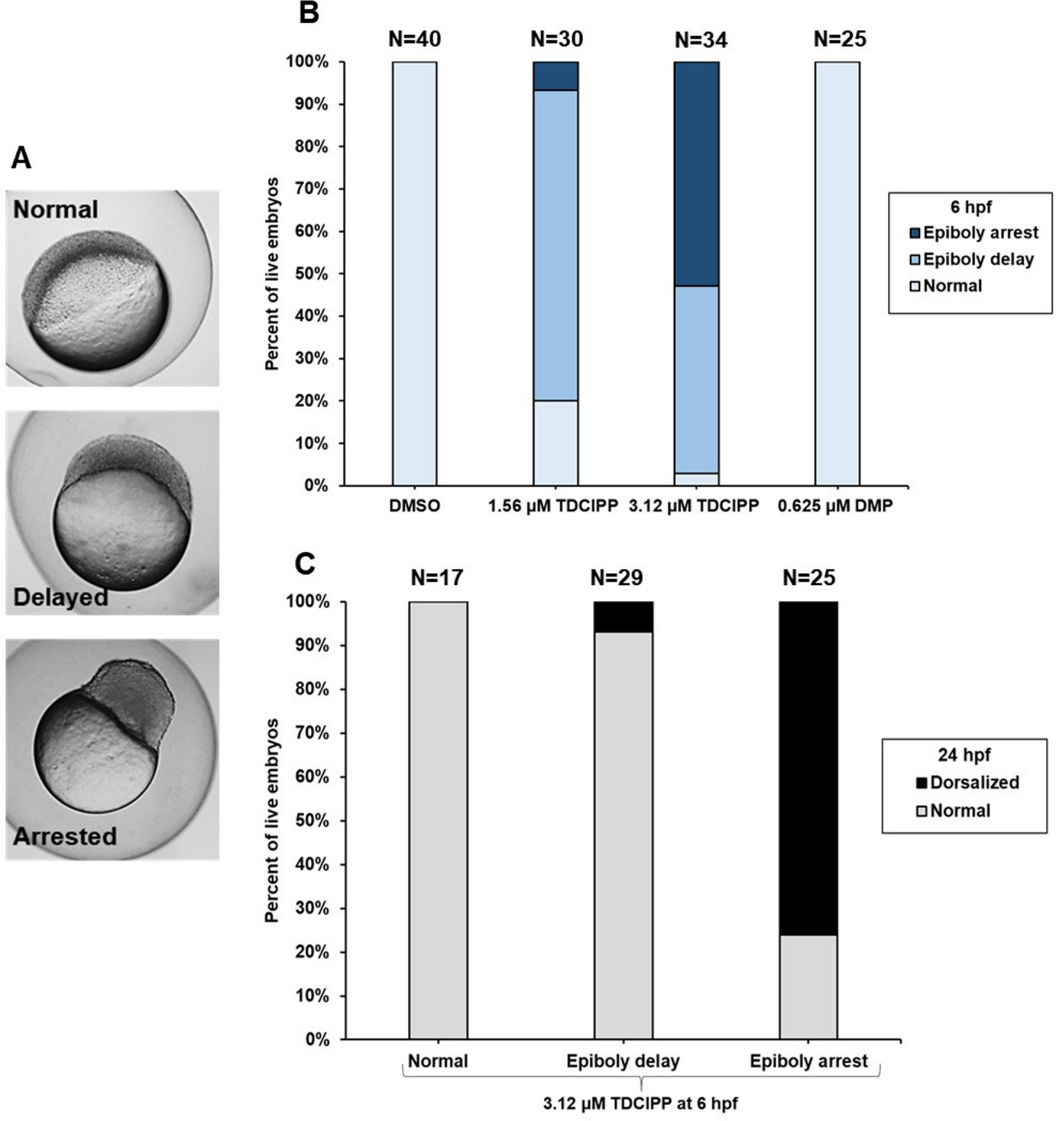
TDCIPP-induced dorsalization at 24 hpf is preceded by epiboly arrest at 6 hpf. (A) Representative images of three epiboly phenotypes (normal, delayed, and arrested) at 6 hpf. Untreated embryos are normally at the germ ring or shield stage by 6 hpf. Epiboly-delayed embryos only progress to the dome and 30% epiboly stage by 6 hpf; epiboly-arrested embryos stall at the high stage by 6 hpf. (B) TDCIPP exposure results in a concentration-dependent increase in epiboly-arrested embryos, whereas DMP-treated embryos show no epiboly defects at 6 hpf. (C) Compared to <10% for normal and delayed embryos, 76% of embryos with epiboly arrest are dorsalized at 24 hpf.

### TDCIPP-induced dorsalization at 24 hpf is preceded by epiboly arrest at 6 hpf

Based on phenotypes observed at 6 hpf (Fig. 3A), we quantified normal, epiboly-delayed, and epiboly-arrested embryos following exposure to TDCIPP (Fig. 3B). While 73% and 6% of embryos were epiboly-delayed and epiboly-arrested, respectively, following exposure to 1.56 µM TDCIPP, 52% of embryos were epiboly-arrested following exposure to 3.12 µM TDCIPP, indicating a concentration-dependent increase in the percentage of epiboly-arrested embryos. Therefore, we isolated normal, epiboly-delayed, and epiboly-arrested embryos following exposure to 3.12 µM TDCIPP at 6 hpf, and continued to expose these groups separately until 24 hpf. Interestingly, while 0% and 7% of normal and epiboly-delayed embryos resulted in dorsalization, 76% of epiboly-arrested embryos resulted in dorsalized phenotypes (Fig. 3C). However, unlike TDCIPP, exposure to 0.625 µM DMP (the highest concentration tested) resulted in no effects on epiboly at 6 hpf (Fig. 3B) despite strong dorsalization effects at 24 hpf (Fig. 3C).

### *szl* and *chd* morphants do not phenocopy TDCIPP-exposed embryos at 6 and 24 hpf

**Figure 4:**
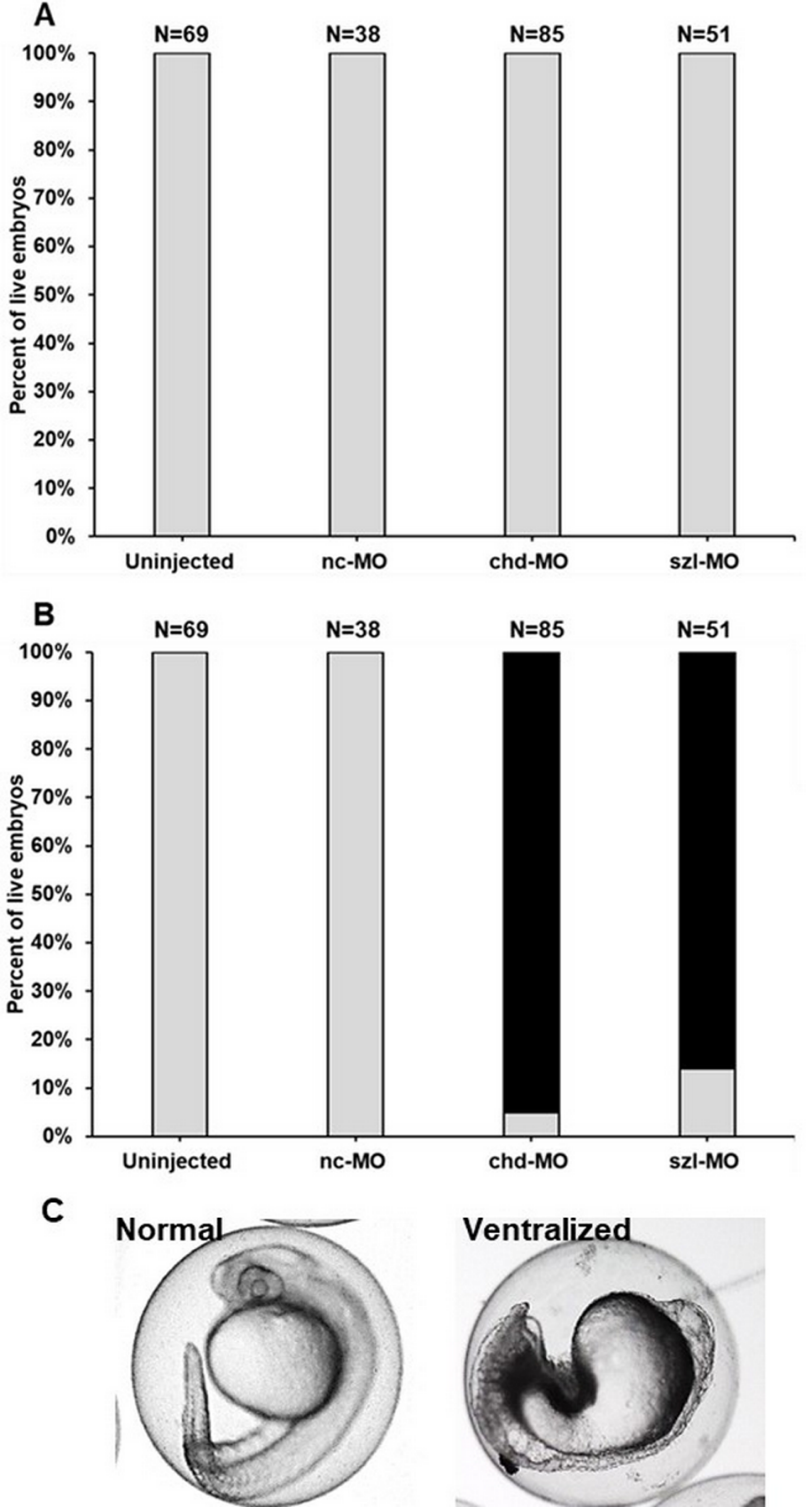
*szl* and *chd* morphants do not phenocopy TDCIPP-exposed embryos at 6 and 24 hpf. (A) Knockdown of *szl* or *chd* results in no effect on epiboly initiation or progression at 6 hpf. Grey bars represent normal embryos. (B) Similar to *chd* morphants (95% ventralized), 84% of *szl* morphants are ventralized at 24 hpf. Grey bars represent normal embryos whereas black bars represent ventralized embryos. (C) Representative images of normal vs. ventralized embryos at 24 hpf. Ventralized embryos are characterized by a decrease in dorsal structures (head, eye, etc.) and expansion of ventral structures.

Based on our microarray data, we relied on a splice-blocking MO to determine whether *szl* knockdown phenocopies TDCIPP-induced epiboly delays at 6 hpf; *chd* MO was used as a positive control. PCR-based determination of *szl* knockdown efficacy is presented in Fig. S2. Interestingly, in contrast to TDCIPP-exposed embryos, knockdown of *szl* (or *chd*) did not affect epiboly at 6 hpf (Fig. 4A). Furthermore, 84% and 95% of *szl* and *chd* morphants, respectively, resulted in strong ventralization at 24 hpf (Fig. 4B).

## Discussion

The current study builds upon our previous work demonstrating that TDCIPP induces significant defects on epiboly and embryonic development (Kupsco et al., 2017; McGee et al., 2012; Volz et al., 2016). Although internal embryonic doses of TDCIPP at 6 hpf (Volz et al., 2016) are likely higher than TDCIPP levels detected in human placental samples (Ding et al., 2016), our study suggests that, if concentrations are elevated *in utero*, TDCIPP has the potential to impact the normal progression and trajectory of early embryonic development. While neither phenotypic (McGee et al., 2012) nor transcriptomic changes (this study) were detected at 2 hpf (pre-MZT), our microarray data revealed that significant changes in gene expression were induced by 6 hpf (post-MZT)—changes that are also associated with impacts on epiboly progression (Kupsco et al., 2017).

BMP signaling plays an important role in ventral cell fate specification within developing zebrafish embryos. Embryos deficient in BMP signaling agonists (e.g., *tolloid* and *bmpr1aa*) are dorsalized, whereas those deficient in BMP antagonists (e.g., *chd*) are ventralized (Langdon & Mullins, 2011; Smith et al., 2011) due to disruptions in dorsoventral patterning. Interestingly, GO enrichment analysis (based on a FDR *p* < 0.1) revealed that BMP signaling and dorsoventral patterning were two of the top biological processes affected by TDCIPP. These two processes included a total of four genes (*bambia, chd, szl,* and *bmpr1aa*), three of which inhibit BMP signaling (*bambia*, *chd*, and *szl*) (Tsang et al., 2000; Langdon & Mullins, 2011) and one of which activates BMP signaling (*bmpr1aa*) (Smith et al., 2011). However, the direction of response for BMP signaling components was inconsistent at 6 hpf (*bambia*, *szl*, and *bmpr1aa* were decreased, whereas *chd* was increased), suggesting that additional studies are need to confirm whether TDCIPP directly targets BMP signaling or impacts other BMP-connected pathways that regulate embryonic development.

Another important biological process that was affected by TDCIPP was somitogenesis, a process that is partially dependent on BMP signaling and plays a crucial role in development following epiboly (Stickney, Barresi & Devoto, 2000). A decrease in *igf2a* and *tbx16* suggests that segmentation and somitogenesis may be impacted later in development (White, Kyle & Wood, 2009; Warga et al., 2013). Among other transcripts that were significantly impacted, *kazald2* belongs to the family of fibroblast growth factors that play a key role in cell migration and embryo patterning (Jovelin et al., 2010) and, similar to a previous study (Fu et al., 2013), a decrease in *kazald2* may be associated with TDCIPP-induced epiboly delays. Overall, our data suggest that TDCIPP exposure may lead to a delay or disruption of early embryonic development via impacts on key biological processes involved in BMP signaling, dorsoventral patterning, and somite formation.

Based on our GO enrichment analysis, we determined whether initiation of TDCIPP exposure at 0.75 hpf resulted in abnormal dorsoventral patterning by 24 hpf. As our data suggested that TDCIPP may disrupt BMP signaling, we used DMP—a known BMP pathway inhibitor (Paul et al., 2008)—as a positive control. Within zebrafish embryos, DMP blocks BMP-mediated SMAD 1/5/8 phosphorylation and downstream gene transcription (Paul et al., 2008), resulting in varying classes of dorsalization by 24 hpf. These classes were originally defined using zebrafish mutants deficient in regulators of BMP signaling activity (Mullins et al., 1996; Paul et al., 2008; Schumacher et al., 2011); therefore, embryos belonging to these classes are genetically deficient in one or more agonists of the BMP signalling pathway. Similar to DMP, TDCIPP exposure resulted in a concentration-dependent increase in the severity of dorsalization, suggesting that TDCIPP exposure results in inhibition of normal BMP signaling and downstream effects on dorsoventral patterning. Moreover, the presence of split yolk phenotypes (while rare) also supports the conclusion that TDCIPP interferes with BMP-related pathways, as these phenotypes are characteristic of zebrafish *split top* mutants deficient in BMP signaling (Langdon et al., 2016).

While quantifying TDCIPP-induced epiboly defects in our previous study (Kupsco et al., 2017), two distinct phenotypes emerged at both concentrations: (1) epiboly-delayed embryos at the dome-30% epiboly stage at 6 hpf, but eventually complete epiboly and (2) epiboly-arrested embryos at the high stage at 6 hpf, but continue developing in the absence of epiboly. Untreated embryos are normally at the germ ring or shield stage by 6 hpf. Within the current study, we discovered that, while the majority of epiboly-delayed embryos appear normal at 24 hpf, 76% of epiboly-arrested embryos resulted in dorsalized phenotypes, suggesting that epiboly arrest—but not epiboly delay—at 6 hpf leads to dorsalization by 24 hpf. Previous studies have shown that mutations in early zygotic genes lead to epiboly defects, disruptions in dorsoventral patterning, and severe deformities in surviving embryos (Haffter et al., 1996; Kane et al., 1996; Kane, McFarland & Warga, 2005; Langdon et al., 2016). Therefore, the link between epiboly arrest and dorsalization observed following TDCIPP exposure suggests that TDCIPP may interfere with the normal function of early zygotic gene products. Interestingly, contrary to TDCIPP, DMP exposure resulted in no effects on epiboly, suggesting that (1) DMP-induced dorsalization at 24 hpf is not associated with epiboly arrest and (2) despite phenotypic similarities with DMP at 24 hpf, TDCIPP-induced dorsalization may be driven by other additional pathways that also lead to epiboly defects.

Among transcripts that were significantly different at 6 hpf, *szl* was the most affected, with a ∼4-fold decrease relative to vehicle controls at 6 hpf. Based on this finding, we relied on a *szl*-specific, slice-blocking MO to determine whether, similar to TDCIPP, a decrease in *szl* results in epiboly defects and/or dorsalization. However, contrary to our hypothesis, *szl* morphants were identical to uninjected and negative control MO embryos at 6 hpf. *szl* is a gene encoding a secreted Frizzled-related protein that mediates ventral cell fate and dorsoventral polarity by inhibiting activity of the BMP signaling gradient and stabilizing Chordin during late-blastula and early gastrula (Muraoka et al., 2006; Yabe et al., 2003). Previous studies with *szl* morphants and *ogon* (*szl*) mutants indicate that decreased Sizzled activity destabilizes Chordin and disrupts BMP signaling, inducing ventralized phenotypes that phenocopy *chd* mutant embryos (Martyn & Schulte-Merker, 2003). Consistent with these studies, 84% and 95% of *szl* and *chd* morphants, respectively, were ventralized at 24 hpf, demonstrating that, similar to *chd*, *szl* knockdown disrupts dorsoventral patterning (leading to ventralization). However, TDCIPP-exposed embryos were strongly dorsalized (and not ventralized), suggesting that, due to the complicated nature of BMP signaling and its interaction with other developmental pathways, the ventralizing effects of decreased *szl* may be insufficient to drive dorsoventral patterning toward ventralization.

## Conclusions

In conclusion, this study provides new insight into the effects of a widely used flame retardant on early embryonic development. Specifically, this study resulted in four major findings: (1) TDCIPP disrupts biological processes that regulate BMP signaling and dorsoventral patterning; (2) TDCIPP-exposed embryos phenocopy embryos exposed to DMP from 0.75 to 24 hpf; (3) TDCIPP-induced epiboly arrest at 6 hpf results in dorsalized embryos at 24 hpf; and (4) *szl* and *chd* morphants do not phenocopy TDCIPP-exposed embryos at 6 or 24 hpf. Future studies are needed to uncover mechanisms underlying TDCIPP-induced epiboly arrest, as well as determine how defects in epiboly lead to irreversible effects on dorsoventral patterning (dorsalization) later in development. Moreover, future studies that rely on SMAD phosphorylation and *in situ* localization of key BMP signaling components are needed to confirm whether TDCIPP is interfering with BMP signaling during early embryonic development.

##  Supplemental Information

10.7717/peerj.4156/supp-1 Supplemental Information 1 Figures S1 and S2

10.7717/peerj.4156/supp-2 Data S1 Raw data supporting Figs. 1–4 and Fig. S1

## Additional Information and Declarations

### Microarray Data Deposition

The following information was supplied regarding the deposition of microarray data: All CEL and CHP files are available for download at NCBI’s GEO DataSets database (Accession ID GSE106875).

### Data Availability

The following information was supplied regarding data availability: The raw data has been provided as a Supplemental File.
