# Supplementary material for: Tris(1,3-dichloro-2-propyl) phosphate disrupts dorsoventral patterning in zebrafish embryos
Source: PeerJ. 2017 Dec 14;5:e4156. doi: 10.7717/peerj.4156 (PMC5733366; doi:10.7717/peerj.4156)
Supplement: Supplemental Information 1 [file peerj-05-4156-s001.pdf]

SUPPLEMENTAL FIGURES

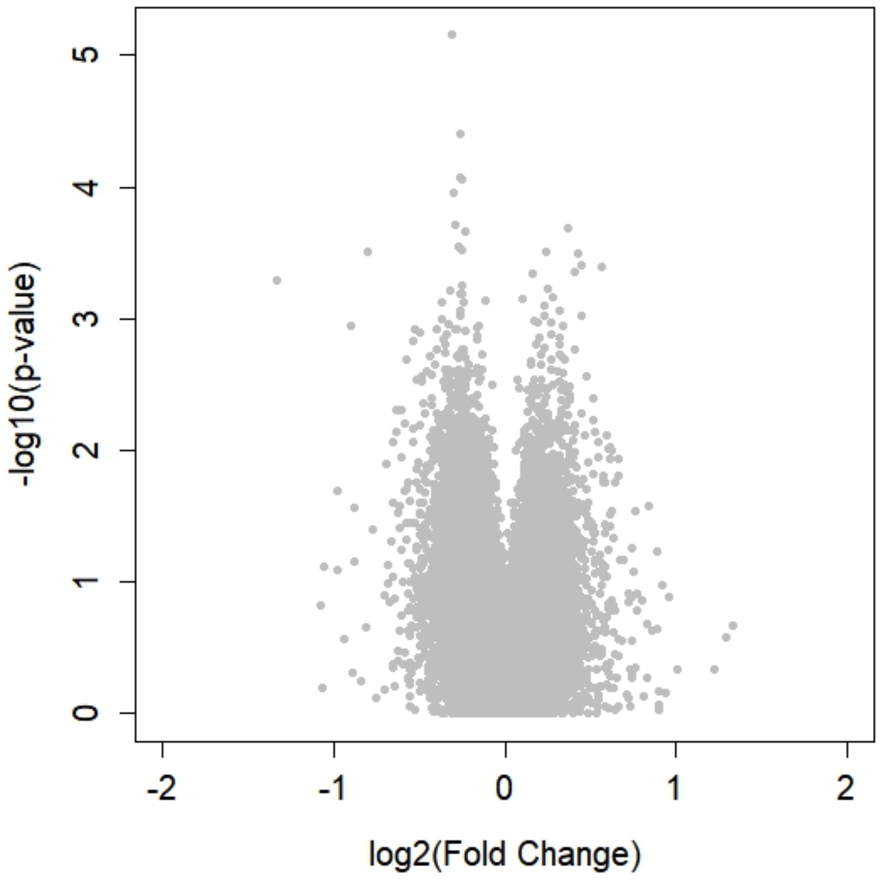

Figure S1: Volcano plot showing transcriptomic responses following exposure to 2 µM TDCIPP from 0.75 to 2 hpf. x axis = log2 (fold-change); y axis = -log10 (p-value). No differentially expressed genes were detected based on a FDR $p < 0.1$.

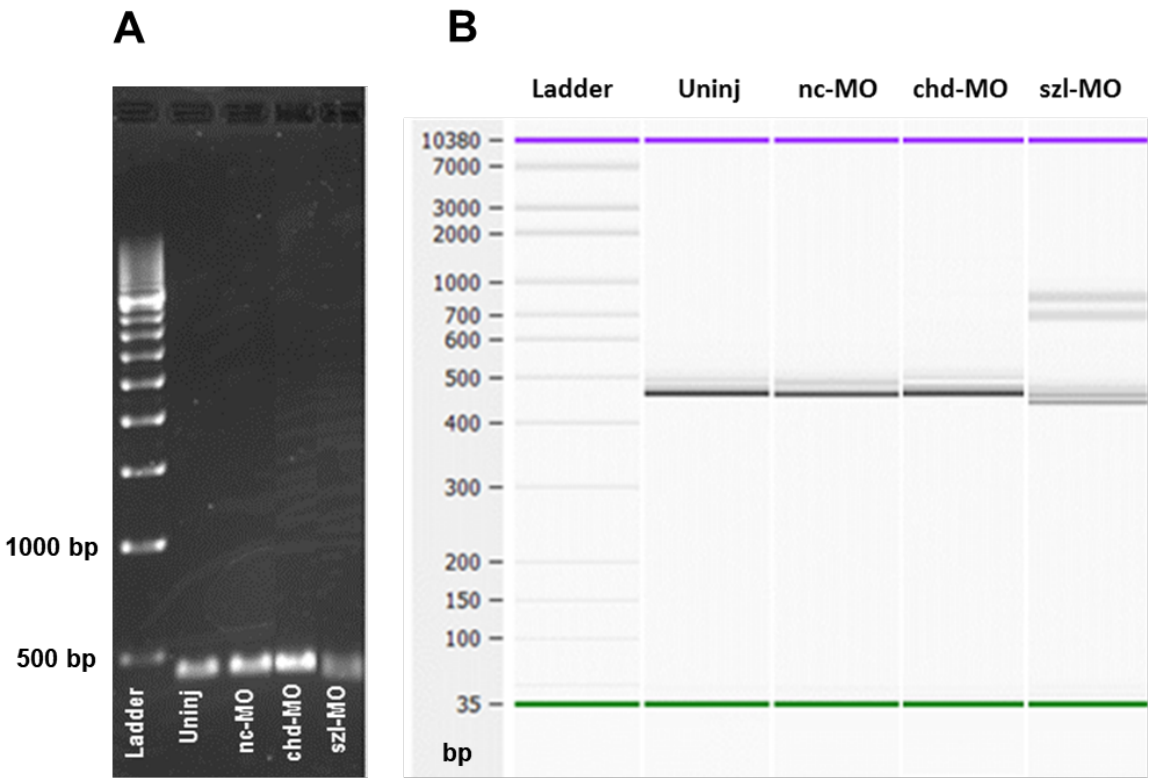

Figure S2: ***szl*** **knockdown results in a decrease in the concentration of spliced** ***szl*** **mRNA.** Agarose gel (left) and Bioanalyzer (right) images of an amplified E1:E2 *szl* fragment (~450-bp) in MO-injected embryos at 6 hpf. While both images show a decrease in the E1:E2 *szl* fragment in szl-MO-injected embryos, two additional bands were detected between 500-1000 bp on the Bioanalyzer that did not appear in the gel. However, a band indicative of intronic (I1) inclusion (~2000 bp) was absent using both approaches.
